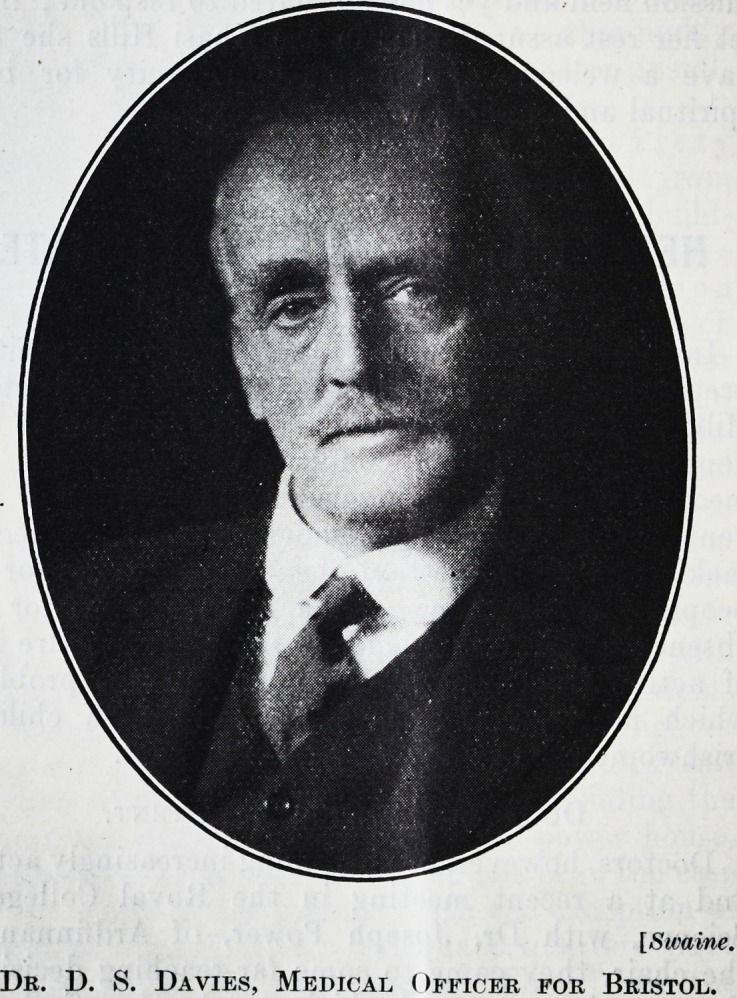# The Public Health: Interviews with Local Authorities: Bristol

**Published:** 1924-04

**Authors:** 


### THE PUBLIC HEALTH.

### INTERVIEWS WITH LOCAL AUTHORITIES.

### XVI.—THE CITY AND COUNTY OF BRISTOL.

The City of Bristol is also an independent county by virtue of a Charter granted by King Edward III. to show his gratitude to the town for aid given towards the invasion of France. A royal visitor of to-day would probably be moved to congratulate this city of 383,000 people, not only on its position as the sixth largest town in England, but also on the fact that its average death-rate and infant mortality rate are lower than those of the other five large towns. The infant mortality rate in particular would be especially worthy of mention; it stood at 62.5 per 1,000 births for 1923, this being the lowest rate ever recorded in this, or possibly any other large city. As to royal visits—other times, other manners. For instance, Henry VII. on a visit fined the townsmen because their wives were too sumptuously apparelled. This and other interesting historical facts were communicated to us by Dr. D. S. Davies, the Medical Officer of Health, who accompanied Mr. H. J. Maggs, the genial Chairman of the Public Health Committee at Bristol, in the interview which these gentlemen courteously accorded to us recently.

[*Veale, Bristol.*

Councillor H. J. Maggs, Chairman of City and County of Bristol Health Committee.

[*Swaine.*

Dr. D. S. Davies, Medical Officer for Bristol.

#### Plague and Pestilence.

It is well that we should sometimes be reminded that our health was not always the subject of the vigilant care of Health Committees and highly skilled Medical Officers. The City of Bristol, for example, has had considerable experience of disaster by plague and pestilence. In 1349 plague is said to have destroyed half the population and grass grew inches high in the streets, which were too narrow for vehicular traffic; projecting upper storeys cut off light and air. In succeeding centuries sweating sickness and plague alternated as causes of serious mortality. The most serious outbreak in the nineteenth century was cholera, which claimed 2,000 victims in 1849. It again visited the city in 1866. Lest we should be complacent about the improved conditions of to-day, there comes a sharp reminder of the grim toll of disease, despite all our endeavours, in the shape of 1,058 deaths from influenza in 1918.

#### A Proud Record of Public Health Work.

In the presence of a serious prevalence of typhus fever, the old Sanitary Committee in Bristol decided on the appointment of a Medical Officer of Health (at first termed "Medical Inspector" of Health), to whom fell the successful dealing with the cholera introduction of 1866. This office was held from 1866 to 1886 by Dr. Davies, the father of the present Medical Officer, who has held that office continuously since that year. This must surely be a unique record of local medical service. During his period of office of thirty-seven years Dr. D. S. Davies has not only witnessed the city increase by some 160,000 people and quadruple its acreage, but has shared in the process of evolution of public health endeavour from the purely environmental supervision of the earlier days, through the beginnings of bacteriological developments and the scientific study of disease causation, to the present-day highly organised, partly preventive, partly curative system aiming at the eradication of special diseases, or dealing with maternity and childhood. With strong epidemiological leanings Dr. Davies accomplished, in the middle of this period, some useful work on milk-carried typhoid fever (Clifton College outbreak) and scarlet fever, and, with Professor Walker Hall, on both fæcal and urinary typhoid carriers. During the war period two introductions of bubonic plague, one into the city, one into the port, and also cerebro-spinal fever and encephalitis lethargica, said Dr. Davies somewhat whimsically, added new interest to the usual routine of home infections.

### Smallpox Control.

Mr. Maggs particularly emphasised the indebtedness of the city to its Medical Officer for the promptness with which he has checked every attempt on the part of smallpox to gain an entry into the town. During his tenure of office there have been as many as 101 such attempts; in the year 1903 there were introductions of this disease, though of a curiously mild type, on fifteen separate occasions.

### The War Against Tuberculosis.

The Chairman said that his Committee have accepted the view of their Medical Officer that tuberculosis is an endemic disease in civilised communities from which there is no escape; and that endeavour is better exercised in dealing with its more amenable evidences in childhood, with some hope of a resulting immunity, than in concentrating only on its later manifestations as declared pulmonary phthisis. Hence a large estate (Frenchay Park) has been purchased for devotion to child-tuberculosis only, to be equipped on the plan of Alton and Hayling Island (both visited by the full Committee) up to 100 beds, a plan hitherto deferred by Treasury difficulties, but shortly, it is hoped, to be overcome. The Bristol Scheme for Tuberculosis, when fully realised, will, it is claimed, be as complete and as well adapted to the actual natural history of tuberculosis as any other scheme in the country.

### Ships in the Heart of the City.

The visitor to Bristol is at once struck with the fact that ships of quite considerable tonnage come right into the heart of the city. Until early in the nineteenth century the tidal river flowed and ebbed to and from the city quays, but in 1803 this 2½ miles of river were dockised and a relief culvert cut. More recently the Avonmouth and Portishead Docks have provided accommodation for the larger steamers, thus avoiding the dangers of the tortuous river. But the presence of a considerable number of ships in the centre of the city itself should serve as a reminder to the citizens to strengthen the hands of the Medical Officer in every way in the important part of his duties which is comprised in his port work. Disease is not infrequently brought by ship to the country. Smallpox has thus been introduced on several occasions. Plague has also been introduced, and various diseases such as malaria and beri-beri have arisen from time to time. Following the cholera at Hamburg in 1892, an inspection launch was on duty for several years and a hospital ship was moored at the mouth of the Avon. It is a matter of some concern to Dr. Davies that this inspection launch and hospital ship are no longer available.

### Water Supply and Sewerage.

It is worth recording that some of the conduits which brought water to the monasteries in the fourteenth century still exist in excellent preservation. The citizens of to-day, through the agency of the Bristol Water Works Company, prefer to receive their supplies from deep springs in the Mendips and deep wells at Chelvey. Large storage reservoirs at Barrow, recently equipped with filter beds, insure a constant service of 22 gallons per head daily. The sources of supply are so far removed from possible sources of contamination, and are so carefully guarded, that this supply may be regarded as one of the most valuable sanitary assets of the city. Bristol is (apart from some recently included outlying portions) a completely sewered city, cesspools are not countenanced, and no dry systems of disposal are in use. The aggregate length of the main sewers is about 150 miles. The sewers at present discharge without treatment into the tidal Avon with double tidal valves at each outlet, but are so designed with regard to capacity, fall and position that they may ultimately be converged on one point from which an outfall sewer may be continued to a suitable point in the Bristol Channel.

### A Milk Crusade.

The Chairman of the Health Committee impressed upon us that in Bristol they are enthusiasts for clean milk. They recognise that in this matter they are dependent on the co-operation of the farmers, and they congratulate themselves that this they have very largely secured. The Farmers' Union with others are represented on the Milk Advisory Committee which has been set up, with very successful results. They have had a "Milk Week," and have issued posters to cowkeepers and dairymen containing sound advice. Mr. Maggs emphasised the fact that the simple precautions necessary to the production of clean milk did not involve any heavy outlay.

### Maternity and Infant Welfare.

Notably good work has been done in this direction under the part-time supervision of Dr. J. C. Heaven. There are five Municipal Ante-Natal Clinics, one Municipal Infant Clinic, and twenty Schools for Mothers and Infant Consultation Centres, which are under voluntary supervision but affiliated to the Bristol Infant Welfare Association and Council of Schools for Mothers, of which the Medical Officer of Health is the President. Dr. Davies especially emphasised the great value of the voluntary workers in this connection.

### Housing.

It is difficult to say much on this subject which has not been said elsewhere. Historically, it is interesting to record that congestion and overcrowding have arisen largely through the building on to small gardens to provide accommodation, before the days of cheap transport, for the workers near their work, rather than through any deliberate and inhuman building of back-to-back houses as in some industrial areas. Three trained Inspectors have recently been appointed for special housing work under the Chief Housing Inspector, and three large areas have been surveyed and reported to the Housing Committee for completion of necessary schemes. These schemes may be subject to reconsideration, if it should appear possible that they may interfere with the vitally important Housing work under the Housing Acts.

### Home Nursing.

Both the Chairman and Dr. Davies had no hesitation in saying that the most important post-war improvement has been the transference of disease inquiry, in the common child infections, such as diphtheria and scarlet fever, from the male Inspectors to Home Nurses, all fully trained, who can carry intimate advice into the homes and to the mothers. The result has hitherto been most encouraging, and this obvious improvement deserves extension. When the nurses were first put on to diphtheria work during the serious epidemic of 1921 they found, amongst other things, the habit of neighbourly visiting by mothers, who were more often than not accompanied by a baby in arms and other small children to see the patient. People, said Dr. Davies, do not grasp the fact that diphtheria and other diseases live in the patient and are caught directly from the patient; they have the mistaken notion that if the drains and sink are all right there is no danger. They have to be patiently taught, and this can only effectively be done by a trained nurse who has the confidence of the mother, and can go into more intimate detail than is possible for a male Inspector. Immediate abatement of the epidemic took place with the arrival of the Home Nurses, and has been maintained. This success implies great saving of life.

### Cordial Relations with General Practitioners.

A discussion arising out of the issue of a Diphtheria Circular to doctors in the city in 1922 led Mr. Maggs to remark that it is very gratifying to the Health Committee that such cordial relations subsist between the Medical Officer and the general practitioners of the city. They are evidently, and very properly, proud of their Medical Officer's work, and he for his part is gratefully appreciative of the support given him by his Committee and the Chairman, Mr. Maggs.

---

### A Record in Hospital Service.

The Board of Management of the West Bromwich and District Hospital at a recent meeting unanimously passed the following resolution:—"That it is with great pleasure that this Board records the fact that Dr. Sansome has been one of the hon. surgeons of this hospital for upwards of twenty-one years, while his beloved father, the late Dr. T. Sansome, served this institution for the long period of forty-six years (as hon. surgeon for thirty-seven years and as consulting surgeon for nine years), a joint period probably unique in the annals of hospital service, and that this Board trusts that Dr. Sansome will be spared for many years to carry on his great work in the cause of sick and suffering humanity."

## Figures and Tables

**Figure f1:**
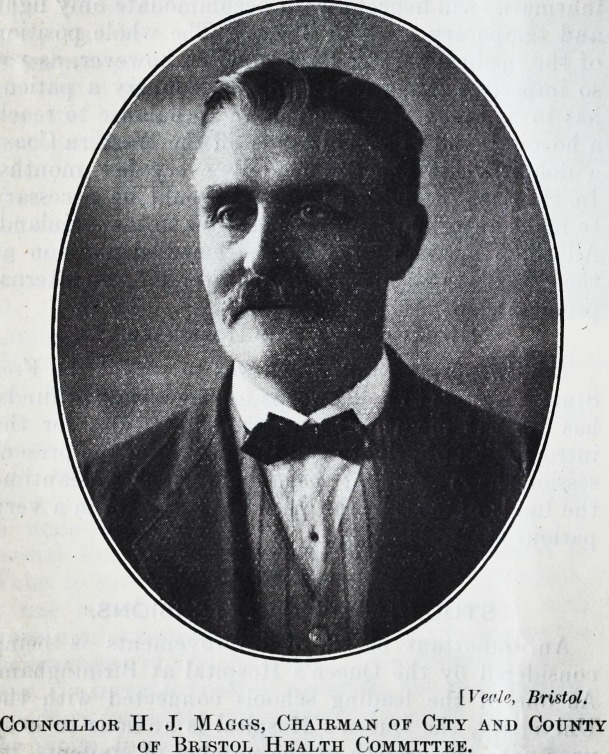


**Figure f2:**